# Microbiota–metabolite axis in endometriosis: Pathogenic mechanisms and clinical implications

**DOI:** 10.7555/JBR.39.20250566

**Published:** 2026-07-28

**Authors:** Moyuan Li, Aiyuan Yue, Lingfeng Gu, Feiyang Li, Nuo Ye, Sujuan Xu, Zhen Gong, Dake Li, Pengfei Xu

**Affiliations:** 1 Department of Gynecology, Women's Hospital of Nanjing Medical University (Nanjing Women and Children's Healthcare Hospital), Nanjing, Jiangsu 210004, China; 2 Nanjing Women and Children's Healthcare Institute, Women's Hospital of Nanjing Medical University (Nanjing Women and Children's Healthcare Hospital), Nanjing, Jiangsu 210004, China; 3 Department of Clinical Laboratory, Women's Hospital of Nanjing Medical University (Nanjing Women and Children's Healthcare Hospital), Nanjing, Jiangsu 210004, China

**Keywords:** endometriosis, microbiota, metabolite

## Abstract

Growing evidence highlights the gut, reproductive tract, and endometrial microbiota as important functional contributors in the pathogenesis of endometriosis (EM). Studies have revealed a characteristic microbial imbalance in patients with EM, marked by a reduced abundance of beneficial bacteria and an enrichment of opportunistic pathogens. These microbial communities are thought to influence disease progression primarily through metabolic activity, as demonstrated by metabolomic studies showing their capacity to modulate host immune and endocrine responses. This imbalance may contribute to several key metabolic disturbances, including decreased levels of short-chain fatty acids, particularly butyrate; a shift in tryptophan metabolism toward the kynurenine pathway; elevated β-glucuronidase activity; increased lipopolysaccharide production; and altered secondary bile acid profiles. Functionally, these metabolic alterations are thought to contribute to EM by disrupting immune homeostasis, enhancing estrogen signaling, and driving systemic inflammation, thereby creating a permissive microenvironment for ectopic lesion growth and invasion. Targeted interventions, such as probiotics, high-fiber dietary strategies, fecal microbiota transplantation, and selective modulation of microbial or host metabolic enzymes, are emerging as promising non-hormonal therapeutic approaches. Nonetheless, further studies incorporating longitudinal cohort designs and integrative multi-omics approaches are essential to establish causality and facilitate the development of precise diagnostic and personalized treatment strategies.

## Introduction

Endometriosis (EM) is defined as the presence of endometrial-like glands and stroma outside the uterine cavity. Clinically, it presents as pelvic pain, infertility, or pelvic masses, affecting approximately 10% of women of reproductive age, with a peak incidence between 25 and 35 years of age^[1]^. Upon estrogen stimulation, ectopic endometrial glands undergo hormone-dependent cyclic changes. Depending on their location, such as the ovaries, fallopian tubes, pelvic peritoneum, or ligaments, they can cause local bleeding, pain, fibrotic nodule formation, or infertility^[2–3]^. The pathogenesis of EM is complex and multifactorial. The most widely accepted theory posits that retrograde menstruation facilitates the translocation of viable endometrial cells to the pelvic cavity and other sites^[4]^. Although some degree of retrograde menstruation occurs in most women, only 1%–2% develop EM, indicating that additional pathophysiological mechanisms are involved^[5]^. Various factors have been implicated in the initiation and progression of EM, including inflammatory cytokines^[6]^, alterations in the immunopathological microenvironment^[7]^, environmental factors^[8]^, and microbiota^[9]^.

In recent years, several studies have suggested a significant role for the microbiota, including the gut, reproductive tract, and tissue-resident microbiota, in the pathogenesis and progression of EM^[9]^. Furthermore, Mendelian randomization analyses have provided novel insights into the potential causal relationship between the gut microbiota and EM. Members of the families Christensenellaceae and Ruminococcaceae, as well as *Eubacterium ruminantium*, exhibit protective associations against EM. Conversely, *Anaerotruncus* and *Olsenella* have been identified as risk-associated taxa that increase susceptibility to EM, whereas taxa such as those in the order Bacillales and the family Prevotellaceae have been implicated in elevated disease risk^[10–12]^. These microbial communities are thought to primarily influence EM progression through their metabolites, which regulate key processes such as local inflammation, estrogen metabolism, and neurogenesis. These findings provide genetic evidence supporting potential causal links between the gut microbiota and EM. Therefore, this review systematically summarizes and integrates current evidence on the roles of the gut, reproductive tract, and tissue-resident microbiota in the development and progression of EM from the dual perspectives of microbial composition and metabolite-mediated mechanisms and explores their potential clinical implications.

## Microbiome characteristics in EM

**Figure 1:**
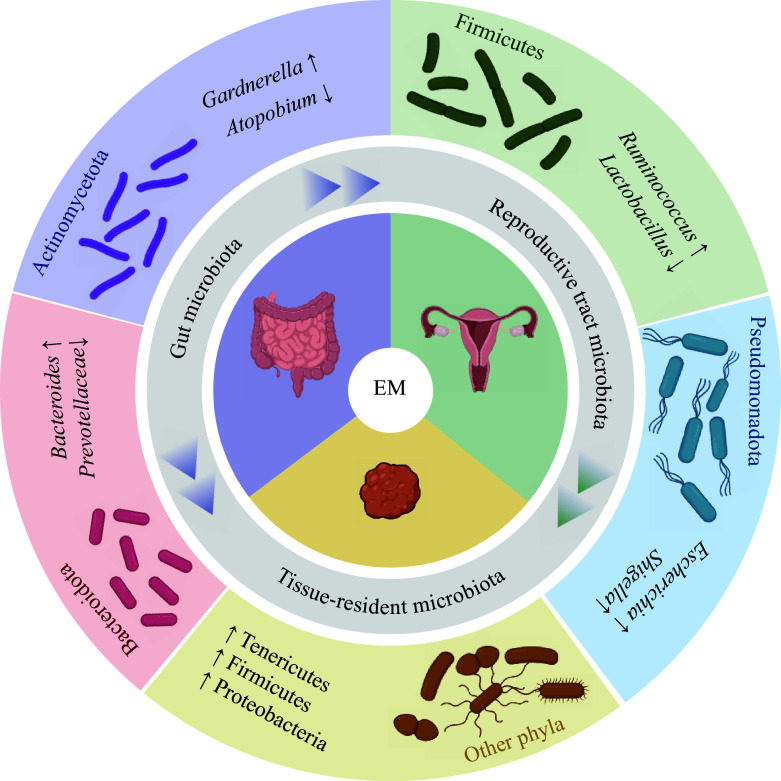
Microbiome characteristics in endometriosis (EM). This circular schematic illustrates the compositional changes and potential inter-compartmental microbial translocation across three anatomical niches in EM. The central circle denotes the disease state (EM), surrounded by three anatomical modules representing distinct microbiota habitats: gut microbiota (purple), reproductive tract microbiota (green), and ectopic tissue microbiota (yellow). Triangular arrows between modules indicate putative migration routes of microorganisms among these compartments. The outermost ring comprises four phylum-level modules and one "Others" module. Each phylum module displays representative genera with directional arrows indicating increased (↑) or decreased (↓) abundance. Created with BioRender.com. Xu, P. (2025) https://BioRender.com/a51kabp.

Dysbiosis, particularly that of the gut microbiota, is associated with conditions such as inflammatory bowel disease^[13]^, arthritis^[14]^, and certain cancers^[15]^, highlighting its role in regulating systemic inflammation *via* immune-mediated mechanisms. Given the importance of aberrant inflammatory responses in EM pathogenesis, a potential microbiota-EM link has been suggested^[16]^. Dysbiosis may elevate systemic estrogen levels by influencing hepatic metabolism and intestinal β-glucuronidase activity^[17]^, thereby promoting the growth of ectopic lesions and amplifying inflammation^[18]^. In addition to the gut, alterations in the local microbiota of the reproductive tract and endometrial tissues have garnered increasing attention in EM research. Patients with EM exhibit reduced vaginal microbiota diversity and decreased relative abundance of *Lactobacillus*^[19]^, accompanied by an increase in the abundance of opportunistic pathogens such as *Gardnerella* and *Prevotella*. This dysbiotic state may promote the adhesion and survival of ectopic endometrial tissues by altering the local immune microenvironment and enhancing the secretion of inflammatory factors^[20]^. The upper female reproductive tract is now recognized as non-sterile, and the microbiota composition of the endometrium and peritoneal fluid exhibits EM-associated alterations^[21]^. For instance, the enrichment of bacterial genera such as *Fusobacterium* in ectopic lesions may directly contribute to lesion establishment and progression by activating growth factors and multiple inflammatory pathways. Furthermore, bacterial translocation within the pelvic cavity, such as through retrograde menstruation or intestinal bacterial translocation, may further interconnect the gut and reproductive tract microenvironments, forming an integrated microbial-immune regulatory network. ***Fig. 1*** illustrates the compositional changes of the microbiome and potential inter-compartmental microbial translocation across the gut, reproductive tract, and ectopic tissue in EM. Based on these multifaceted associations, the following sections systematically explore the characteristics of these microbial alterations.

### Gut microbiota

The gut microbiota composition of patients with EM differs significantly from that of healthy individuals, a phenomenon consistently observed in both human clinical samples and animal models^[22–26]^. In human studies, comparative analyses of fecal microbiomes from patients with EM and healthy controls have revealed significant differences in the abundance of at least 12 bacterial genera^[23]^. Animal studies provide complementary evidence. For instance, in a rhesus macaque EM model, distinct alterations were observed in the shed fecal microbial profiles of affected individuals^[26]^. In mouse models of EM, although some studies using α- and β-diversity analyses did not show significant changes in overall community diversity, higher-resolution taxonomic analyses revealed specific compositional and structural alterations in the gut microbiota^[22,24]^. These findings support the conclusion that EM is associated with disease-specific gut microbial dysbiosis.

The dysbiosis observed in EM does not manifest as a global, unidirectional shift across bacterial taxa but presents a heterogeneous and context-dependent pattern. At the phylum level, reported trends are highly variable, potentially due to differences in experimental models, host species or strains, dietary composition, and analytical methodologies. For example, one mouse study identified an enrichment of the phyla Firmicutes and Actinobacteria in the EM group, accompanied by a significantly elevated Firmicutes-to-Bacteroidetes ratio, a metric frequently regarded as an indicator of gut dysbiosis^[22,27]^. In contrast, another study reported increased Bacteroidetes abundance concomitant with reduced Firmicutes in EM mice^[28]^. These two studies employed different modeling approaches (intraperitoneal injection of endometrial fragments versus surgical autotransplantation combined with antibiotic intervention), which may substantially influence microbial outcomes.

At the genus level, the changes were more pronounced and taxonomically specific. The relative abundance of several taxa with putative protective or homeostatic functions was reduced. For example, a depletion of *Lactobacillus* was observed in rhesus macaques and some human cohorts^[23,26]^, and lower levels of *Alloprevotella* and *Turicibacter* were found in patients with EM^[23]^. Conversely, the abundance of taxa associated with pro-inflammatory signaling and disease progression increased. Representative taxa include a higher prevalence of *Shigella/Escherichia*-dominated communities in women with EM^[25,29]^, enrichment of Gram-negative bacteria such as *Escherichia coli* (*E. coli*) and *Enterobacter* in a rhesus macaque model^[26]^, and elevated levels of Ruminococcaceae UCG-014, *Bifidobacterium*, and *Parasutterella* in murine EM models^[22]^. Taxon-specific microbial alterations are summarized in ***Table 1***.

**Table 1:** Characteristics of differentially abundant microbiota in endometriosis (EM)

Phyla	Bacterial name/Group	Gram stain	Abundance change (EM *vs.* control)	References
Gut microbiota				
Actinomycetota	*Bifidobacterium*	G^+^	Up	[22]
Bacteroidota	Bacteroidales S24-7 group	G^−^	Up	[22]
	*Bacteroides*	G^−^	Up	[23]
	*Alloprevotella*	G^−^	Down	[23]
	Prevotellaceae UCG-001 group	G^−^	Up	[22]
	*Parabacteroides*	G^−^	Up	[23]
Firmicutes	*Ruminobacter*	G^+^	Down	[23]
	*Coprococcus*	G^+^	Up	[23]
	Lachnospiraceae NK4A136 group	G^+^	Up	[22]
	*Oscillibacter*	G^+^	Up	[23]
	Ruminococcaceae UCG-014 group	G^+^	Up	[22]
	*Lactobacillus*	G^+^	Down	[23,26]
	*Turicibacter*	G^+^	Down	[23]
Pseudomonadota	*Parasutterella*	G^−^	Up	[22]
	Unclassified Enterobacteriaceae	Typically G^−^	Up	[23]
	*Shigella/Escherichia*	G^−^	Up	[25,29]
	*Escherichia coli*	G^−^	Up	[26]
	*Enterobacter*	G^−^	Up	[26]
Reproductive tract microbiota				
Actinomycetota	*Gardnerella*	variable	Up	[19,25,34]
	*Atopobium*	G^+^	Down	[25]
	*Corynebacterium*	G^+^	Up	[25,32]
Bacteroidota	*Prevotella*	G^−^	Up	[19]
Firmicutes	*Lactobacillus* spp.	G^+^	Down	[19]
	*Streptococcus*	G^+^	Up	[25,32,34]
	*Enterococcus*	G^+^	Up	[34]
	Veillonellaceae (family)	G^−^	Up	[19,25]
Pseudomonadota	*Escherichia coli/Shigella*	G^−^	Up	[25,29,34]
	Enterobacteriaceae (family)	G^−^	Up	[25,32]
	*Pseudomonas*	G^−^	Up	[25,32]
Tenericutes	*Ureaplasma urealyticum*	G^−^	Up	[19,25]
Tissue-resident microbiota				
Fusobacteria	*Fusobacterium nucleatum*	G^−^	Up	[38]
			Indifferent	[42]
Firmicutes	*Faecalibacterium prausnitzii*	G^+^	Up	[39]
Proteobacteria	*Pseudomonas*	G^−^	Up (predominant in ascites)	[41]

These distinct microbial alterations may contribute to the pathogenesis and progression of EM *via* multiple interrelated mechanisms. Shifts in gut microbiota composition are closely associated with perturbations in estrogen metabolism. Microbes, such as those belonging to the phyla Firmicutes and Bacteroidetes and the genus *Bifidobacterium*, possess genes encoding β-glucuronidase, an enzyme that catalyzes the deconjugation of estrogens and facilitates their reabsorption into the systemic circulation. Consequently, microbial dysbiosis may lead to elevated circulating bioactive estrogen levels, thereby establishing a permissive hormonal milieu that supports the persistence, growth, and progression of endometriotic lesions^[17]^. Furthermore, the enrichment of pro-inflammatory bacteria (*Shigella* and *Escherichia*) may amplify systemic or local inflammation, whereas the depletion of protective commensal taxa may compromise immune regulatory capacity and inflammatory resolution^[25–26,29]^. Anatomical factors, such as a shorter anogenital distance, may modulate these processes by increasing the likelihood of bacterial translocation between anatomical compartments^[30]^. Collectively, the gut microbiota has emerged as a critical determinant of EM pathophysiology and represents a promising target for dietary modulation and microbiota-based therapeutic interventions^[27]^.

### Reproductive tract microbiota

Studies on the lower reproductive tract microbiome in patients with EM have revealed a heterogeneous and nuanced microbial landscape. Some studies have reported no significant differences in the cervix between patients with EM and healthy controls^[31]^, whereas others have identified specific microbial perturbations. The most consistent finding was a marked reduction in protective *Lactobacillus* species, which are essential for preserving a healthy, acidic vaginal environment and cervicovaginal microbial homeostasis^[19]^. At the phylum level, this dysbiosis often corresponds to a decreased relative abundance of Firmicutes (to which *Lactobacillus* spp. belong) and an increased abundance of other phyla such as Actinobacteria and Proteobacteria. Concurrently, there is a relative enrichment of a broad spectrum of genera, many of which are associated with bacterial vaginosis (BV) or pro-inflammatory states. These include members of the family Enterobacteriaceae, the genera *Streptococcus*, *Pseudomonas*, and *Corynebacterium*^[25,32]^, the family Veillonellaceae, the genus *Gardnerella*, and the species *E. coli/Shigella* and *Ureaplasma urealyticum*^[19,25]^. Interestingly, one study reported the absence of *Atopobium*, a genus associated with BV, in the cervical and vaginal microbiota of patients with EM^[25]^. In contrast, other reports indicate that non-*Lactobacillus*-dominant microbial communities, particularly those enriched in *Gardnerella* and *Atopobium*, frequently colonize the cervicovaginal epithelium in women with BV^[33]^. These seemingly conflicting findings suggest that the lower reproductive tract microbiota in EM may exhibit context-dependent or subtype-specific dysbiosis patterns. Collectively, these observations indicate that the lower reproductive tract in patients with EM exists in a state of disease-associated microbial imbalance, which may contribute to disease progression through modulation of local immune responses and inflammatory signaling.

The existence of distinct endometrial microbiota remains controversial, as microbial detection may partially reflect contamination by cervicovaginal or intestinal bacteria. Studies have reported an increased abundance of *Gardnerella*, *Streptococcus*, *Enterococcus*, and *E. coli* in the endometrium of patients with EM^[34]^, which have previously been associated with menopause and estrogen-depleted states^[35]^. As *Enterococcus* and *E. coli* are common gut colonizers, these findings raise the possibility of intestinal bacterial carryover or contamination in certain samples. However, other investigations have yielded contrasting results, identifying an enrichment of *Prevotella*, Veillonellaceae, and *Atopobium* in the endometrium of women with EM; these taxa are frequently detected in women with BV^[19]^. Such inconsistencies likely reflect disease heterogeneity, methodological variability, or differences in disease stage or sampling strategies.

In summary, the current evidence indicates a higher prevalence of BV-associated bacteria in the cervicovaginal and endometrial microbiota of women with EM. Functionally, this shift reflects the depletion of protective *Lactobacillus* alongside an enrichment of pro-inflammatory taxa (*Gardnerella* and *E. coli*), which may facilitate disease progression by maintaining a chronic inflammatory microenvironment that favors ectopic lesion establishment. Despite inter-study variability in specific bacterial compositions, certain BV-associated microbiota have consistently been associated with EM.

### Tissue-resident microbiota

In recent years, microbial communities in pathological tissues have attracted increasing attention. In human tumor tissues, bacteria have been identified as intratumoral residents within both cancer and infiltrating immune cells^[36]^. *Fusobacterium*, a common commensal bacterium found in the oral cavity and gastrointestinal tract, is considered an opportunistic pathogen. Species within the genus *Fusobacterium*, such as *Fusobacterium nucleatum* (*F. nucleatum*), participate in the development of periodontitis and may facilitate tumorigenic processes by eliciting inflammatory cytokines such as interleukin-6, interleukin-8, and tumor necrosis factor^[37]^. The presence of tissue-resident microbiota within tumors has prompted renewed interest in understanding how microbes may influence non-malignant tumor-like pathological conditions. Despite its histologically benign nature, EM shares several hallmark features with neoplastic diseases, including sustained proliferation, resistance to apoptosis, local invasiveness, angiogenesis, and immune evasion. These tumor-like characteristics suggest that ectopic endometrial lesions may provide a permissive niche for microbial colonization or persistence, analogous to that observed in malignant tissues.

Emerging evidence indicates that the endometrium and ectopic lesions of patients with EM show significant enrichment of *F. nucleatum*^[38]^ and *Faecalibacterium prausnitzii*^[39]^ compared with healthy controls. Among these, *F. nucleatum* activates transforming growth factor beta (TGF-β) signaling, thereby promoting the transformation of fibroblasts into myofibroblasts with enhanced proliferative, adhesive, and migratory capabilities, ultimately contributing to the progression of endometrial lesions^[38]^. Under certain conditions, this enrichment may be associated with vaginal dysbiosis^[40]^. Accordingly, these bacteria may gain access to the upper female reproductive tract *via* mechanisms such as retrograde menstruation from the dysbiotic vaginal microenvironment, leading to the colonization of ectopic lesions. Furthermore, one study reported that *Pseudomonas* is the predominant bacterial genus in the peritoneal fluid of patients with EM, with approximately 95% originating from the intestine^[41]^. This phenomenon is often accompanied by elevated lipopolysaccharide (LPS) levels and disruption of intestinal barrier integrity. Through fecal microbiota transplantation in a mouse model, researchers experimentally demonstrated the translocation process of bacteria along the "gut-peritoneal cavity-lesion" axis, providing mechanistic evidence for the intestinal origin of the microbiota within the ectopic endometrium^[41]^.

However, a recent study revealed no significant difference in the presence of *Fusobacterium* spp. in the normal endometrium between patients with EM and healthy individuals^[42]^. This contradiction with previous findings is likely due to the heterogeneity of the study populations. Moreover, differences in host factors, such as geographical location, dietary habits, and ethnic background, may account for the divergent conclusions regarding *F. nucleatum* enrichment, suggesting that its potential diagnostic or prognostic utility in EM warrants further investigation.

## Microbiota-derived metabolites in EM

Following the identification of dysbiosis in the gut, reproductive tract, and ectopic tissue microbiota in EM, a central question arises: How do these microbial perturbations influence host physiology? Women with EM exhibit gut dysbiosis and significant differences in stool metabolites^[43]^. The microbiota functions as an active, metabolically integrated biological unit that generates metabolites that enter the systemic circulation and modulate immune, endocrine, and metabolic processes. Therefore, metabolomics, which enables the comprehensive profiling of small-molecule metabolites, provides critical insights into upstream microbial-host interactions^[44]^. Accordingly, microbial dysbiosis in EM likely drives significant remodeling of the host metabolite landscape.

In the context of EM, gut microbiota imbalance may lead to pronounced changes in microbiota-derived metabolites, which primarily fall into three categories. First, dietary components are abnormally transformed by gut microbes. For example, short-chain fatty acids (SCFAs), including acetate, propionate, and butyrate, are major fermentation products of undigested dietary fibers generated by colonic bacteria and play essential roles in maintaining intestinal homeostasis^[45]^. In EM, reduced SCFA levels may impair anti-inflammatory and immunoregulatory functions, thereby promoting a pro-inflammatory microenvironment conducive to lesion establishment and growth. Tryptophan (Trp) metabolism in the gastrointestinal tract is highly dependent on the microbiota, which converts Trp into various bioactive compounds^[46]^. Altered Trp metabolites in EM can modulate immune responses and affect pain perception, thereby eliciting neurological symptoms associated with the disease. Second, host-synthesized molecules undergo microbial modification in the gut. A key example is secondary bile acids (SBAs). Primary bile acids produced in the liver are converted into SBAs, such as deoxycholic acid (DCA) and lithocholic acid (LCA), by specific gut bacteria (*Clostridium* spp.) through dehydroxylation reactions, a process tightly regulated by the microbial composition^[47]^. In EM, SBAs may contribute to inflammation and cellular proliferation, potentially exacerbating the development of ectopic lesions. Third, certain metabolites originate directly from the microorganisms themselves. LPS, a major component of the outer membrane of Gram-negative bacteria, can translocate into the circulation during dysbiosis and act as a potent inflammatory activator, triggering systemic inflammation *via* Toll-like receptor 4 (TLR4) signaling^[48]^. Elevated LPS levels in EM are believed to drive chronic pelvic inflammation and pain while promoting the adhesion and invasion of endometrial cells. Simultaneously, β-glucuronidase produced by certain gut bacteria hydrolyzes conjugated estrogen into its free, biologically active form, enabling its reabsorption and disrupting normal enterohepatic estrogen cycling^[49]^. The microbial reactivation of estrogen in EM increases local and systemic estrogen levels, which are key factors in stimulating endometrial tissue growth and survival outside the uterus.

**Figure 2:**
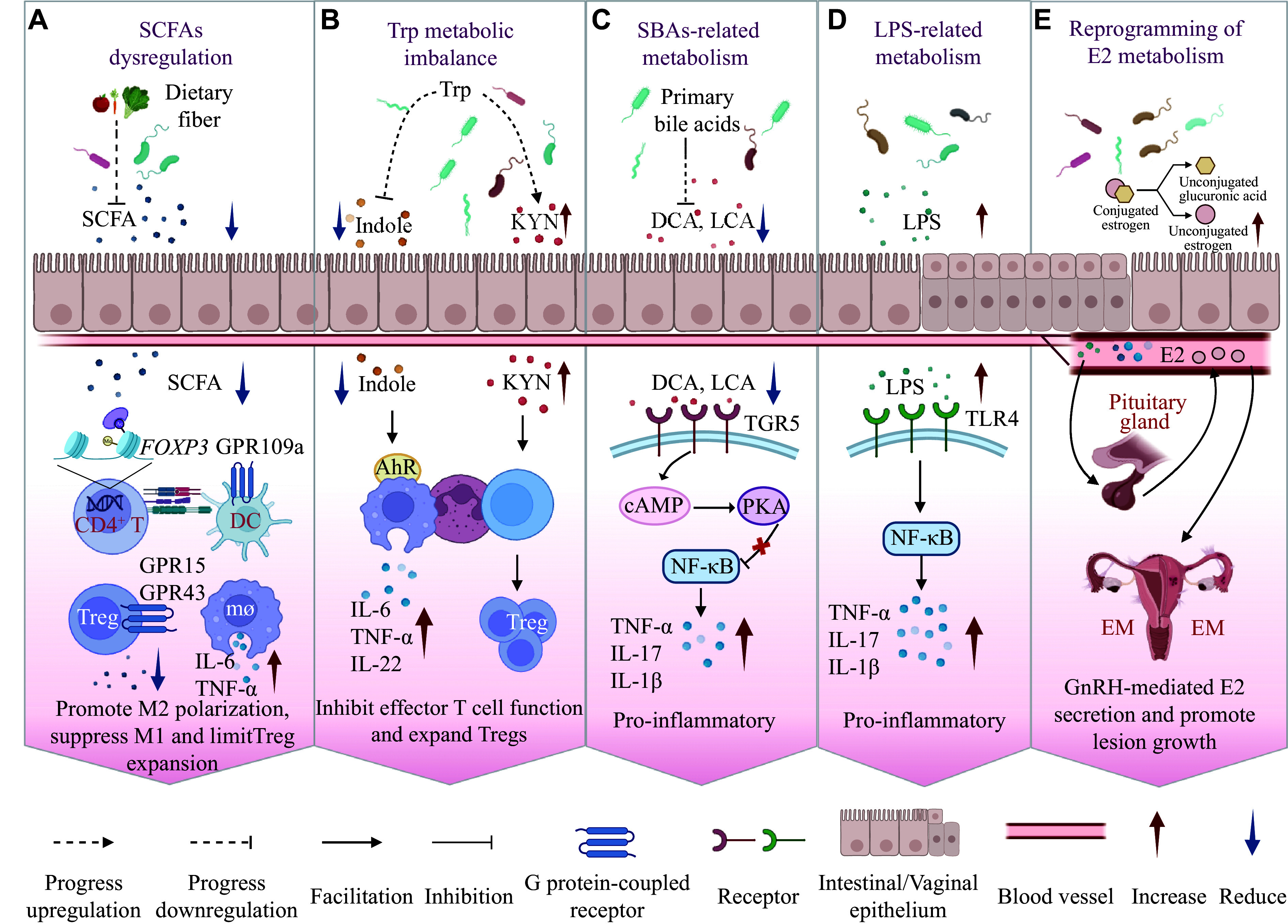
Proposed mechanisms supported by current experimental evidence. This schematic illustrates five interconnected pathways through which microbial imbalance disrupts host metabolism, immune function, and estrogen homeostasis in EM. Panels are arranged horizontally. A: SCFA dysregulation: Reduced SCFA-producing bacteria decrease SCFAs, impairing Treg/M2 polarization and enhancing NF-κB-mediated inflammation. B: Trp metabolic aberration: Altered microbiota shifts tryptophan toward the kynurenine pathway, expanding Tregs while suppressing effector T cells. Reduced AhR ligands (IPA, IAA) increase IL-6/TNF-α, fostering immune tolerance. C: SBA-related metabolic alteration: Reduced SBA-producing bacteria lower DCA/LCA, diminishing TGR5-cAMP/PKA signaling, which disinhibits NF-κB and promotes pro-inflammatory cytokines. D: LPS-related metabolic perturbation: Gram-negative bacterial overgrowth elevates LPS, activating NF-κB *via* TLR4 and driving TNF-α/IL-17/IL-1β secretion, contributing to chronic pelvic inflammation. E: Reprogramming of estrogen metabolic imbalance: Microbial β-glucuronidase increases free E2 reabsorption, elevating local/systemic estrogen. Altered neuroactive metabolites disrupt the hypothalamic-pituitary-ovarian axis, creating a self-reinforcing cycle of estrogen excess and inflammation. Collectively, these dysbiosis-driven metabolic shifts are proposed to promote ectopic lesion survival, proliferation, and invasiveness in EM. Abbreviations: AhR, aryl hydrocarbon receptor; DC, dendritic cell; DCA, deoxycholic acid; E2, estradiol; EM, endometriosis; IAA, indole-3-acetic acid; IL, interleukin; IPA, indole-3-propionic acid; KYN, kynurenine; LCA, lithocholic acid; LPS, lipopolysaccharide; mø, macrophage; NF-κB, nuclear factor-kappa B; SBAs, secondary bile acids; SCFAs, short-chain fatty acids; TGR5, Takeda G protein-coupled receptor 5; TLR4, Toll-like receptor 4; TNF-α, tumor necrosis factor alpha; Treg, regulatory T cell; Trp, tryptophan. Created with BioRender.com. Xu, P. (2025) https://BioRender.com/a51kabp.

These three dysbiosis-driven metabolic alterations constitute the critical biochemical basis for EM. Hence, we propose the microbiota–metabolite axis as a pathological framework to systematically elucidate the mechanisms underlying dysbiosis in the gut and vaginal microbiota through the modulation of specific metabolic pathways, thereby reshaping local and systemic immune microenvironments and promoting the initiation and progression of EM. In the following section, drawing on the five metabolic reprogramming pathways (SCFAs, Trp, SBAs, LPS, and estrogen metabolism) outlined in ***Fig. 2***, we explore the mechanisms of microbiota-derived metabolites in EM from the perspective of altered metabolic profiles.

### SCFA dysregulation

The production of SCFAs is critically dependent on the composition of the gut microbiota. Key SCFA-producing genera include *Bacteroides*, *Bifidobacterium*, *Ruminococcus*, *Lactobacillus*, *Faecalibacterium*, *Prevotella*, and *Eubacterium*^[50]^. In the context of EM, a dysbiotic state characterized by a reduced abundance of specific beneficial taxa, particularly *Ruminococcus* and *Eubacterium* species, is the primary cause of impaired SCFA generation. Beneficial bacteria, such as *Eubacterium ruminantium* (family Ruminococcaceae), are positively correlated with SCFA production in EM^[51]^, and species such as *Ruminococcus* *faecis* play a pivotal role in butyrogenesis^[52]^.

SCFAs, principally acetate (60%), propionate (20%), and butyrate (20%), are produced by the gut microbiota through anaerobic fermentation of dietary fibers^[45]^. These potent SCFAs exert systemic immunomodulatory effects through distinct mechanisms. Their primary immunoregulatory actions are mediated by activating specific G protein-coupled receptors (GPCRs) and inhibiting histone deacetylases (HDACs)^[53]^. Butyrate, the main energy source for colonocytes^[45]^ and recognized for its anti-inflammatory and anticancer properties^[54–55]^, is considered as a key regulator of colonic regulatory T cell (Treg) generation by directly promoting histone acetylation at the *FOXP3* locus to drive naïve CD4^+^ T cells toward a Treg phenotype^[56]^ or by indirectly inducing Treg differentiation *via* dendritic cell activation through GPR109a^[57]^. Furthermore, butyrate enhances Treg function and FOXP3 expression by inhibiting HDACs (HDAC9)^[58]^, thereby promoting FOXP3^+^ Treg expansion^[59]^. Propionate and acetate recruit extraintestinal Tregs to the colon *via* GPR15 and GPR43, respectively^[60]^. Beyond Tregs, SCFAs suppress nuclear factor (NF)-κB-mediated inflammatory responses in macrophages^[61]^ and may promote macrophage polarization toward the anti-inflammatory M2 phenotype over the pro-inflammatory M1 phenotype.

SCFA deficiency resulting from gut dysbiosis plays a significant role in the pathogenesis of EM. The consequent reduction in systemic anti-inflammatory capacity and compromised immunoregulation creates a permissive environment for ectopic lesions. Decreased butyrate levels stemming from a reduced abundance of key producers weaken the suppression of NF-κB-mediated inflammation^[61]^ and impair Treg differentiation and function^[56,62]^. One study revealed that experimental depletion of Tregs in mice exacerbated lesion growth and inflammation^[63]^. Moreover, butyrate supplementation in mouse models significantly reduced the endometriotic lesion burden^[58,64]^. Butyrate exerts bidirectional regulation of steroidogenesis, and its deficiency may impair the correction of the hyperestrogenic state in EM^[65]^. Altogether, gut dysbiosis-induced SCFA deficiency impairs systemic anti-inflammatory capacity and intestinal barrier integrity, thereby facilitating immune imbalance, the diffusion of inflammatory mediators, and the establishment and persistence of ectopic endometrial tissue^[66]^.

### Aberrations in tryptophan metabolism

The dysbiotic gut microbiota profile is a key driver of the metabolic imbalance of Trp in EM. Dysbiosis is characterized by reduced levels of *Lactobacillus* and *Bacillus* species and an increase in *E. coli*. This reduction affects known commensal bacteria capable of producing aryl hydrocarbon receptor (AhR) ligands, and these beneficial species are primarily limited to *Lactobacillus* species^[67]^ and *Peptostreptococcus russellii*^[68]^. Concurrently, the increased abundance of *E. coli*, which, along with some *Lactobacillus* species, increases tryptophanase expression to convert Trp into indole^[69]^, thereby redirecting the metabolic flux. The decrease in the number of *Bacillus* species that generate tryptamine *via* decarboxylation^[70]^ further contributes to the loss of bioactive molecules. This dysbiosis is reflected by reduced levels of the bacterial metabolite 4-hydroxyindole (4HI) in feces, which correlates with specific microbial shifts: positively with beneficial genera such as *Faecalibacterium* and the family Lachnospiraceae and negatively with the commensal bacterium *Dorea*^[43]^. This specific microbial shift fundamentally disrupts the homeostasis of host Trp metabolism.

Trp metabolism in the gastrointestinal tract occurs mainly through three pathways^[46]^: (1) direct conversion of Trp by the gut microbiota into AhR ligands and other bioactive molecules; (2) catabolism *via* indoleamine 2,3-dioxygenase 1 (IDO-1) in immune and epithelial cells, forming the kynurenine (KYN) pathway; and (3) synthesis of serotonin by enterochromaffin cells. Microbial metabolism is the primary source of AhR activation in the gut^[71]^. During microbial tryptophan metabolism, the oxidation and reduction pathways yield indole-3-acetic acid (IAA) and indole-3-propionic acid (IPA), both of which modulate intestinal permeability and immune function^[72]^. Indole, a key intermediate metabolite, acts as an interspecies signaling molecule that influences bacterial physiology^[73]^. Thus, by shaping Trp flux across these pathways, gut microbes critically regulate host barrier integrity, local immunity, and systemic physiology.

In EM, dysbiosis-driven disruption manifests as a critical imbalance between the KYN and indole pathways, which play a decisive role in disease progression. The altered microbiota specifically enhances the KYN pathway while suppressing the indole pathway. The KYN pathway is markedly upregulated in patients with EM due to elevated IDO-1 activity, leading to increased KYN levels. KYN may promote immune tolerance by suppressing effector T cells and expanding Tregs, thereby facilitating the immune evasion of ectopic lesions^[74]^. In contrast, the microbial indole pathway was suppressed. Fecal metabolomic analyses revealed significantly reduced levels of beneficial metabolites, such as IPA. As a key AhR ligand, IPA exerts anti-inflammatory and antioxidant effects by activating this receptor and inhibiting the production of pro-inflammatory factors, such as IL-6 and TNF-α, thereby contributing to its anti-inflammatory role^[75]^. The decrease in the levels of other immunomodulatory derivatives, such as IAA, indole-3-lactic acid, and 4HI, further weakens host defense^[43]^. Quinic acid is a key fecal metabolite that may promote lesion growth in EM^[76]^. This dual alteration, an enhanced immunosuppressive KYN pathway and a deficient protective AhR pathway, generates an immunotolerant microenvironment conducive to lesion survival and proliferation.

The functional significance of AhR signaling in EM is supported by direct cellular evidence at multiple levels. Mechanistically, AhR is expressed in mast cells that infiltrate endometriotic lesions; upon AhR activation, these mast cells produce IL-17 and reactive oxygen species, contributing to a pro-inflammatory microenvironment that sustains lesion development^[77]^. Furthermore, AhR may interact with other receptors, including classical estrogen receptors, thereby modulating the course of estrogen-dependent diseases, such as EM^[78]^. Environmental AhR ligands, such as 2,3,7,8-tetrachlorodibenzo-*p*-dioxin, exert their toxic effects by binding to AhR, forming an activated heterodimer with transcriptional activity that may promote an inflammatory state and facilitate menstrual processes relevant to EM pathogenesis^[79]^. These findings provide a direct mechanistic link between tryptophan metabolic disturbances and EM pathology.

Accordingly, therapeutic strategies targeting the Trp-AhR axis, such as probiotics, prebiotics, IDO-1 inhibitors, and AhR modulators, are emerging as promising approaches for microbiota-based immune modulation in EM^[80]^.

### Secondary bile acid-related metabolic alterations

The production of SBAs in the intestine is directly governed by the gut microbiota. Primary bile acids are converted into SBAs, such as DCA and LCA, through microbial transformations, particularly dehydroxylation, mediated by bacteria such as *Clostridium difficile*^[47]^. DCA and LCA are the most prevalent SBAs. The abundance of specific beneficial bacteria determines SBA levels. A previous study indicated a significant positive correlation between *Ruminococcus* abundance and SBA levels^[66]^, whereas *Eubacterium* species have been shown to regulate bile acid metabolism and mitigate inflammation^[51]^. Therefore, dysbiosis, which involves a decline in the abundance of these key genera, is the primary cause of reduced SBA production.

As important signaling molecules, SBAs can systemically modulate immune balance and inflammatory responses by significantly reducing the production of multiple inflammatory chemokines and cytokines, including TNF-α and IL-17^[81]^. Their potent anti-inflammatory effects are predominantly mediated by the activation of Takeda G protein-coupled receptor 5 (TGR5). TGR5 inhibits NF-κB activation by modulating the cAMP/PKA signaling pathway. For instance, in a murine model of *Staphylococcus aureus*-induced endometritis, DCA exerts protective effects by significantly upregulating TGR5 and PKA expression; this effect is abolished by TGR5 or PKA inhibitors, which block the production of TNF-α and IL-1β and suppress NF-κB activation^[82]^. LCA and DCA suppress pro-inflammatory cytokine production by human peripheral blood-derived macrophages *via *TGR5 activation^[83]^.

In the context of EM, gut dysbiosis-induced SBA deficiency creates a permissive pro-inflammatory milieu that fuels disease progression. Reduced abundance of beneficial bacteria, such as *Ruminococcus* and *Eubacterium*, leads to decreased SBA levels. This deficiency impairs the vital TGR5-mediated suppression of the NF-κB pathway and the production of pro-inflammatory cytokines (TNF-α, IL-1β, and IL-17)^[81–83]^. The consequent loss of systemic anti-inflammatory signaling disrupts immune homeostasis, allowing unchecked inflammation that promotes the survival, implantation, and growth of ectopic endometrial tissue. Direct evidence from human endometriotic stromal cells supports the protective role of TGR5 activation. Activation of TGR5 by its specific agonist INT-777 protects endometriotic stromal cells from TNF-α-induced inflammation and oxidative stress, reducing the levels of pro-inflammatory cytokines (IL-6, IL-8, and MCP-1), adhesion molecules (ICAM-1 and VCAM-1), and NADPH oxidase 4 (NOX4) expression. These effects are mediated through the inhibition of JNK/AP-1 and NF-κB signaling^[84]^. Thus, dysbiosis-driven suppression of SBA production represents a key mechanistic link between gut microbiota alterations and the inflammatory pathogenesis of EM.

### LPS and endotoxemia-related metabolic perturbations

LPS, a major component of the outer membrane of Gram-negative bacteria, acts as a potent inflammatory activator by binding to TLR4 on immune cells, thereby triggering downstream signaling pathways, such as NF-κB. This cascade activates macrophages and induces the release of pro-inflammatory cytokines, including IL-6 and TNF-α^[48]^. In EM, an increased abundance of Gram-negative bacteria, such as those from the family Prevotellaceae, in the gut or reproductive tract, leads to elevated systemic LPS levels. Sustained endotoxemia drives chronic inflammation in the pelvic and systemic compartments, creating a favorable microenvironment for the adhesion, proliferation, and progression of ectopic endometrial cells^[85]^. In the context of EM, LPS enhances TLR4 expression and activates the NF-κB pathway, exacerbating peritoneal inflammation and promoting the development of endometriosis-like lesions in mouse models^[86]^. Moreover, menstrual blood is frequently contaminated with high levels of *E. coli*, and microbial colonization within the endometrium has been documented, both of which may serve as significant sources of systemic LPS and contribute to endotoxemia^[87]^.

### Reprogramming of estrogen metabolic imbalance

Estrogen plays a pivotal role in the pathogenesis of EM, and its homeostasis is regulated by the gut microbiota. First, during gut dysbiosis, which is characterized by reduced microbial diversity, microbe-derived β-glucuronidase is abnormally upregulated. This enzyme deconjugates estrogen, which is inactivated by hepatic glucuronidation, and converts it back to its biologically active free form^[49]^. These reactivated estrogens are reabsorbed *via* the enterohepatic circulation, leading to abnormally elevated circulating estrogen levels, which directly promote the growth, shedding, and inflammatory activity of ectopic endometrial tissue^[87]^.

Second, gut dysbiosis can influence the function of the hypothalamic–pituitary–ovarian axis by altering neuroactive metabolites. Dysbiosis-induced changes in the metabolome can elevate the circulating levels of neurotransmitter-like substances, such as serotonin, glutamate, SCFAs, and γ-aminobutyric acid. These metabolites can cross the blood–brain barrier and act on various receptors, including those expressed on gonadotropin-releasing hormone neurons, triggering a cascade of hormonal signals that may lead to increased ovarian estrogen secretion^[9]^. Furthermore, shifts in microbial composition can modulate the production of SCFAs and LPS in response to estrogen imbalance, whereas fluctuations in estrogen levels can reciprocally influence microbial community structure. This bidirectional interaction contributes to the altered immune microenvironment observed in patients with EM^[49,88]^.

### Other metabolic alterations

In addition to the metabolites discussed previously, many other metabolites are associated with alterations in the gut microbiota in EM. Using correlation analysis, a mouse model study confirmed that L-methionine and L-cysteine levels were increased in the feces of mice with adenomyosis. Their abundance is negatively correlated with that of *Bacteroides* but positively correlated with *Desulfovibrio*, suggesting a statistical association between specific amino acid metabolism and microbiota composition^[89]^. Furthermore, other studies using the same disease model have depicted a broader landscape of metabolic dysregulation, identifying the differential expression of various metabolites, including quinic acid, cytosine, and L-methylhistidine, in the feces of affected mice^[76]^. Among these, quinic acid levels increased significantly. Functional experiments confirmed the ability of quinic acid to effectively promote the proliferation of immortalized human endometrial epithelial cells, providing a potential molecular explanation for the direct involvement of microbial metabolites in the growth of ectopic lesions^[76]^. The effects of this metabolic reprogramming were evident in lipid and primary bile acid metabolism. For instance, in the feces of EM model mice, the abundance of bile acids, such as chenodeoxycholic and ursodeoxycholic acids, is increased, whereas the levels of alpha-linolenic acid and its metabolite 12,13-epoxyoctadecatrienoic acid are decreased. This shift may lead to significant perturbations in enriched pathways, including secondary bile acid biosynthesis and alpha-linolenic acid metabolism^[90]^. Altered abundance of specific metabolites has been observed in the fecal samples of women with EM. These biomarkers include linoleic acid, adenine, cytosine, and adenosine (***Table 2***)^[43]^. Although these studies illustrate extensive alterations in the gut microbial metabolic network under disease conditions, most analyses remain descriptive and have yet to elucidate the causal relationship between microbial changes and specific alterations in metabolite levels.

**Table 2:** Differentially abundant microbiota-derived metabolites in endometriosis (EM)

Metabolites	Category	Change in EM (abundance)	References
12,13-Epoxyoctadecatrienoic acid	Fatty acid derivative	Down	[87]
1-Methylhistidine	Amino acid derivative	Down	[76]
20-Deoxyadenosine	Nucleoside	Down	[43]
4-Hydroxyindole	Tryptophan metabolite (indole pathway)	Down	[43]
Acetate	Short-chain fatty acid	Down	[43,45,66]
Adenine	Nucleobase	Down	[43]
Adenosine	Nucleoside	Down	[43]
Alpha-linolenic acid	Polyunsaturated fatty acid	Down	[87]
Butyrate	Short-chain fatty acid	Down	[43,45,51–52,66]
Chenodeoxycholic acid	Primary bile acid	Up	[87]
Cytosine	Nucleobase	Down	[43,76]
Deoxycholic acid (DCA)	Secondary bile acid	Down	[47,66,78–80]
Free (deconjugated) estrogen	Steroid hormone	Up	[49,83]
Indole-3-acetic acid (IAA)	Tryptophan metabolite (indole pathway)	Down	[43,72]
Indole-3-lactic acid (ILA)	Tryptophan metabolite (indole pathway)	Down	[43]
Indole-3-propionic acid (IPA)	Tryptophan metabolite (indole pathway)	Down	[43,75]
Kynurenine	Tryptophan metabolite (Kynurenine pathway)	Up	[74]
L-cysteine	Amino acid	Up	[86]
Linoleic acid	Polyunsaturated fatty acid	Down	[43]
Lipopolysaccharide (LPS)	Bacterial endotoxin	Up	[48,81–83]
Lithocholic acid (LCA)	Secondary bile acid	Down	[47,66,78,80]
L-methionine	Amino acid	Up	[86]
N-formyl-L-methionine	Amino acid derivative	Up	[43]
Propionate	Short-chain fatty acid	Down	[43,45,66]
Quinic acid	Cyclitol-derived metabolite	Up	[76]
Ursodeoxycholic acid	Primary bile acid	Up	[87]

## Clinical applications of microbiota and their metabolites in EM

Our proposed "microbiota–metabolite" axis is supported by accumulating evidence, providing a systematic framework for understanding the underlying mechanisms of EM. This model highlights how microbial dysbiosis acts synergistically across multiple levels, including immune regulation, inflammatory responses, and hormonal balance, by driving disturbances in major metabolic pathways to promote disease development and persistence. Given the central role of the "microbiota–metabolite" axis in EM pathogenesis, the development of novel diagnostic and therapeutic strategies targeting this axis has emerged as a promising research direction. These translational approaches aim to address the critical unmet needs in current clinical practice, namely delayed diagnosis and limited treatment options.

### Diagnostic strategies

Currently, the gold standard for diagnosing EM is invasive laparoscopic surgery, which has an average diagnostic delay of 7–10 years^[91]^. Although most biomarkers are still in the exploratory stage, metabolomics and microbiome profiling offer a robust foundation for developing noninvasive or minimally invasive liquid biopsy approaches. Fecal microbiome-based diagnostic methods have been successfully used in the clinical diagnosis of various diseases^[92]^, suggesting that fecal microbial markers have the potential for cross-disease diagnostic applications.

Multiple studies have indicated that microbial alterations can serve as potential diagnostic targets. *F. nucleatum* infiltration is detectable in the endometrium of 64% of patients with EM^[38]^, suggesting its potential as a biomarker for the auxiliary diagnosis of the disease^[93]^. Importantly, the diagnostic value of this bacterium has been confirmed in colorectal and gastric cancers, with area under the curve (AUC) values of 0.80^[94]^ and 0.813^[95]^, respectively, providing strong evidence for its application in EM diagnosis. Fecal microbial markers also demonstrate diagnostic potential. The ratio of *Prevotella* to *Bacteroides* in the gut is significantly higher in EM patients compared with controls^[96]^. The ratio is known as an important biomarker for obesity, and its alteration in EM further supports its utility as a non-specific indicator reflecting the disease-associated microenvironment for auxiliary diagnosis^[27]^.

Beyond direct microbial markers, microbe-derived metabolites have emerged as promising candidates for non-invasive diagnostic tools. Diagnostic approaches based on blood, urine, and stool-derived metabolites for EM have achieved AUC values exceeding 0.8^[43]^. Multiple untargeted metabolomics studies have consistently identified distinct metabolic signatures in the blood of EM patients^[97]^, involving disturbances in amino acid, lipid, and energy metabolism pathways. Notably, combined metabolite panels have shown excellent performance. Studies have confirmed that a diagnostic model incorporating N-[1H-indol-3-ylacetyl]valine (odds ratio [OR] = 1.601, a tryptophan metabolite) and hippuric acid (OR = 0.368, derived from gut microbial metabolism of dietary components) along with other features achieved an AUC greater than 0.9^[98]^. These metabolic alterations likely reflect the functional output of microbial dysbiosis. Animal studies further suggest the diagnostic potential of related metabolites. Research has found significant differences in fecal metabolites between EM model mice and healthy controls, with quinic acid, lactic acid, and N-acetyl aspartic acid showing elevated levels^[76]^. These differential metabolites hold promise as candidate targets for non-invasive EM diagnosis.

Although microbe-derived markers show great promise for non-invasive EM diagnosis, cautious advancement toward clinical application is needed. Current findings exhibit some heterogeneity, and certain discoveries are based solely on animal models. Therefore, large-scale, multicenter independent cohort studies are still required to systematically validate the diagnostic performance of existing markers.

### Therapeutic strategies

Modulating the "microbiota–metabolite" axis opens up a new frontier for potentially safer and less toxic adjunctive therapies for EM. The following sections provide an overview of these therapies and their potential use in treating EM.

#### Probiotic interventions

Future therapeutic strategies for EM will move beyond generic probiotic supplementation toward precision nutrition tailored to the individual microbial and metabolic profiles. For instance, in patients with low fecal butyrate levels, the targeted administration of butyrate-producing strains (*Clostridium butyricum*)^[99]^ or prebiotics rich in fructooligosaccharides and inulin may be more effective than conventional *Lactobacillus*-based products. Supplementation with *Bifidobacterium bifidum* for 4 weeks modulated the gut microbiota in healthy adults, reducing the abundance of Prevotellaceae and *Prevotella* while increasing that of Ruminococcaceae and Lachnospiraceae, along with elevating butyrate concentrations^[100]^. Animal models have provided proof-of-concept evidence that specific probiotics can reduce lesion size and alleviate pain-related behaviors in EM mice^[101]^.

#### Candidate metabolite-based interventions

Targeting the gut microbiota–metabolite axis represents a novel therapeutic approach for EM, with the direct supplementation of key metabolites demonstrating significant potential. First, butyrate (sodium butyrate) has been shown to effectively inhibit lesion growth in animal models by suppressing endometrial cell survival through multiple pathways, including activation of GPCRs, inhibition of HDACs, and upregulation of the GTPase-activating protein RAP1GAP^[64]^. Second, bile acid metabolites have regulatory value; chenodeoxycholic acid and its derivatives may alleviate EM pathology by modulating the activity of the associated metabolic enzymes^[102]^. Furthermore, the microbial Trp metabolite 4-HI is found at reduced levels in patients with EM, and its supplementation inhibits disease progression, underscoring its potential as a therapeutic candidate^[43]^. In summary, interventions targeting butyrate, bile acids, and Trp metabolites offer diverse and promising avenues for the metabolic management of EM.

#### Dietary modifications

Encouraging patients with EM to adopt a high-fiber, omega-3 fatty acid-rich Mediterranean diet^[103]^ is a safe, cost-effective, and multibeneficial strategy. High-fiber diets are fermented by the gut microbiota into SCFAs, which exert direct anti-inflammatory effects, whereas omega-3 fatty acids are potent natural anti-inflammatory agents^[104]^. Observational studies have identified associations between dietary patterns and EM risk^[105]^, although large-scale randomized controlled trials are required to provide high-level evidence. Moreover, cruciferous vegetables supply indole-3-carbinol, which is converted in the acidic gastric environment into various bioactive compounds—including diindolylmethane^[106]^—that modulate estrogen metabolism and exhibit anti-inflammatory effects, potentially restricting the progression of EM.

#### Fecal microbiota transplantation

As the most radical method of microbial ecosystem reconstruction, fecal microbiota transplantation (FMT) has strong theoretical potential for patients with refractory EM who fail conventional treatments and exhibit severe dysbiosis^[107]^. For example, beneficial bacteria that could be transplanted include Lachnospiraceae, which produces SCFAs^[50]^; *Lactobacillus* species capable of synthesizing AhR ligands^[67]^; and *Clostridium difficile*, which is involved in the conversion of SBAs^[47]^. However, the application of FMT in EM remains entirely theoretical, and no clinical studies have evaluated its safety or efficacy. First, despite rigorous donor screening, potential risks include the transmission of pathogens (multidrug-resistant organisms) and adverse events, ranging from transient abdominal discomfort and diarrhea to rare severe infections^[108–109]^. Second, ethical considerations, including informed consent procedures, privacy protection, and sample ownership, raise concerns, with most survey respondents expressing skepticism^[110]^. Third, FMT products vary widely in their preparation methods, donor selection criteria, and administration routes, and currently lack regulatory approval for non-*Clostridioides difficile* infection indications^[108]^. Consequently, FMT for EM must first be evaluated in well-designed early-phase clinical trials (Phase Ⅰ/Ⅱ) to thoroughly assess its safety and preliminary efficacy before any clinical application and should not be attempted empirically.

## Conclusions

EM has long been viewed as a localized gynecological condition; however, growing evidence positions it within a broader systemic framework centered on the "microbiota–metabolite" axis. This review integrates current findings to reveal a consistent pathological pattern in EM: depletion of beneficial microbes (butyrate producers such as *Ruminococcus* and *Eubacterium*), overgrowth of pro-inflammatory taxa, reduced SCFA levels, a shift in Trp metabolism toward the immunosuppressive KYN pathway, increased β-glucuronidase activity driving estrogen reactivation, and elevated LPS release that may promote inflammation. These alterations disrupt immune homeostasis, enhance estrogenic stimulation, and foster a chronic inflammatory milieu that supports the persistence and progression of ectopic lesions.

However, despite these advances, several key challenges remain. Few findings from existing animal models have been translated into clinical applications in humans. The limitations of the animal models discussed in this study include exogenous estrogen failing to mimic the natural human cycle, interspecies immune differences hindering mechanistic translation, surgical transplantation deviating from the natural course of retrograde menstruation, and disparities in baseline microbial abundances between animals and humans, limiting the extrapolation of metabolic findings. Other key challenges remain, particularly in establishing causality and overcoming the heterogeneity of studies. Therefore, future efforts should focus on developing standardized protocols; integrating multi-omics data; conducting multi-regional, multi-cohort longitudinal studies; advancing lesion-based stratification studies that correlate microbial and metabolic profiles with EM phenotypes to lay the groundwork for personalized diagnostics and treatments; and designing metabolite intervention trials to validate the feasibility of using microbiota-derived metabolites as therapeutic targets.

Well-designed clinical trials testing microbiota-targeted interventions are essential for translating this paradigm into effective diagnostic and therapeutic strategies. Viewing EM through the lens of host–microbiota metabolic crosstalk provides a more integrated understanding of disease pathogenesis and offers new opportunities for biomarker discovery and precision therapeutics.
